# Surgical Exposure Technique for Volar Locking Plate Fixation of Distal Radius Fractures in Patients with Flexor Carpi Radialis Brevis Muscle Anomaly

**DOI:** 10.1155/2021/4512843

**Published:** 2021-10-23

**Authors:** Hiroshi Ninomiya, Makito Watanabe, Kazunari Kamimura

**Affiliations:** ^1^Department of Orthopedic Surgery, Tachikawa General Hospital, Niigata, Japan; ^2^Department of Orthopedic Surgery, Tsuruoka Municipal Shonai Hospital, Yamagata, Japan

## Abstract

The flexor carpi radialis brevis (FCRB) muscle, considered a rare anomaly, is not well known among orthopedic surgeons. The indications for volar locking plates to treat distal radius fractures have recently expanded, and, as a result, encounters with the FCRB are becoming more common. However, few studies have described how to retract an FCRB. Here, we describe seven of 264 patients with FCRB who underwent surgery for distal radius fractures. In one case, the retracted FCRB interfered with the internal fixation. The presented cases demonstrate that the radial retraction of an FCRB with a large muscle belly enables favorable exposure of the distal radius.

## 1. Introduction

The flexor carpi radialis brevis (FCRB) is a relatively rare anomalous muscle of the volar distal forearm. The FCRB originates between the origin of the FPL and the insertion of the PQ; it then runs on the radial side of the FPL and inserts into the second or third metacarpal and the radial side of the carpal bones. It has been described in several anatomical studies and has an incidence of 0.9–7.5% [1–3]. However, reports on FCRB in clinical settings are rare, and many have focused on tenosynovitis of the FCRB [4, 5] or incidental FCRB discovery during dissection of the distal radius [6, 7]. Unlike other anomalous muscles, the FCRB is located on the radial side of the forearm and runs outside the carpal tunnel; therefore, Peers and Kaplan surmised that the FCRB alone does not cause any neurological symptoms [4]. The FCRB is encountered in 2.7–8.7% of surgeries for distal radial fractures [8–12]. Surgeons must be aware of this anomalous muscle when performing surgery for distal radius fractures. However, few studies have described how to retract the FCRB [13]. We encountered seven patients with FCRB among 264 patients who underwent surgery for distal radius fractures. The objectives of this report are when to retract the FCRB ulnarly and when to retract it radially and when to resect its fibers during surgical exposure and volar plate fixation of distal radius fractures.

## 2. Case Presentation

Between 2010 and 2019, 264 patients with distal radius fractures were treated using the volar flexor carpi radialis (FCR) approach and volar locking plates. In seven of those cases, we observed during surgery the FCRB on the radial side of the flexor pollicis longus (FPL) tendon and the superficial layer of the pronator quadratus (PQ) muscle. In these cases, we examined the origin of the FCRB, size of the muscle belly, hypoplasia of the PQ, and impairments of internal fixation. The study was approved by the Tachikawa General Hospital Ethics Organization (approval number: 18008), and informed consent was obtained from all patients.

**Figure 1:**
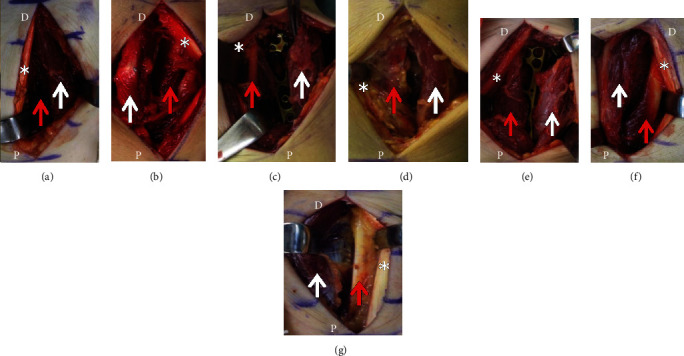
Intraoperative photographs: (a) case 1; (b) case 2; (c) case 3; (d) case 4; (e) case 5; (f) case 6; (g) case 7. D: distal limit of the incision; P: proximal limit of the incision; white arrow: flexor carpi radialis brevis; red arrow: flexor pollicis longus; asterisk: flexor carpi radialis tendon.

**Figure 2:**
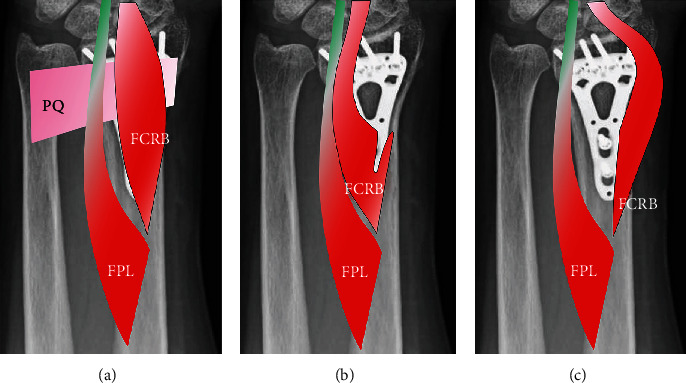
Illustration of the volar aspect of a distal forearm with a flexor carpi radialis brevis (FCRB) muscle: (a) before exposure; (b) FCRB retracted ulnarly and partially detached from the radius; (c) FCRB retracted radially.

The 58 men and 206 women with distal radius fractures had a mean age of 65 years (range, 18–89 years). The three men and four women with FCRB had a mean age of 49 years (range, 24–64 years; Table 1). In all seven patients, the FCRB originated between the origin of the FPL and the insertion of the PQ and ran on the radial side of the FCR and the FPL. The insertion of the FCRB was unknown because it was more distal than the approach described in this report. In all seven cases, the muscle belly of the FCRB was larger than that of the FPL in the operative field (Figure 1). In five cases (cases 2, 3, 5, 6, and 7), the muscle belly of the FCRB was approximately 1.5 times larger than that of the FPL. In these cases, the origin of the FCRB extended to the distal radius metaphysis, shifting the insertion of the PQ from the radial margin of the radius to the center and resulting in hypoplasia of the PQ muscle belly. In case 1, we visualized the fracture site by retracting the FCRB to the ulnar side; in case 2, first, we retracted FCRB ulnarly, but the muscle belly interfered with the insertion of a locking screw on the distal ulnar side, and the FCRB was then retracted radially. In cases 3-7, the FCRB was retracted radially and the muscle belly did not interfere with locking screw insertion (Figure 2). However, in cases 3 and 5, the FCRB originated from the radius and the flexor digitorum superficialis (FDS), thus requiring resection of the muscle fibers that crossed over.

## 3. Discussion

The indications for volar locking plates for the treatment of distal radius fractures have recently increased. As a result, clinical reports on FCRB are becoming more common. Five reports have described FCRB muscles discovered during surgery for distal radius fractures [8–12]. These studies reported an FCRB incidence of 2.7–8.7%. Similarly, we observed an FCRB incidence of 2.6% (seven of 264 cases).

Several case reports have described how to fully expose the distal radius in the presence of FCRB. The first report, described by Kang et al. [6], Lee et al. [10], and Hosokawa et al. [12] retracted the FCRB muscle radially during surgery for distal radius fractures, and they found that FCRB did not hinder internal fixation of the distal radius fractures in any case. Other authors successfully retracted the FCRB muscle to the ulnar side [11, 13]. Laugharne and Power stated that the FCRB made the exposure of the distal radius more difficult, and that distal release was necessary to visualize the fracture site [14]. Werntz et al. showed that the FCRB was resected because its retraction to expose and reduce the fracture site was difficult [15].

Nagata et al. classified FCRB muscles as tendon or muscle belly types [11], and all of our cases were of the muscle belly type. In case 1, ulnar retraction of the small muscle belly of the FCRB did not interfere with fracture exposure nor the insertion of locking screws. In cases 2–7, ulnar retraction of the FCRB and screw insertion were predicted to be difficult because of the large muscle belly; therefore, radial retraction of the FCRB was performed to fully expose the volar metaphysis of the distal radius and insertion of locking screws. Based on our findings, we conclude that FCRB muscle belly size is important. We recommended that when surgeons encounter the FCRB during the volar approach to distal radius fracture fixation, retraction of an FCRB with a small muscle belly to the ulnar side does not hinder the surgical approach or internal fixation, and its radial retraction is recommended when the FCRB has a large muscle belly. However, retraction of the muscle fibers is only indicated when the FCRB originated from the radius and the FDS and crossed over the distal radius.

## 4. Conclusion

The FCRB muscle is not a rare occurrence, so orthopedic surgeons must be aware of its possible presence when performing volar plate fixation of distal radius fractures. The cases presented here demonstrate that radial retraction of an FCRB with a large muscle belly exposes the entire volar metaphysis of the distal radius.

## Figures and Tables

**Table 1:** Clinical data of the seven patients with FCRB.

Case no.	Age (y)	Sex	Side	FCRB origin	PQ status	FCRB size	FCRB retraction
1	63	Female	Right	Distal to FPL origin	Normal	FCRB > FPL	Ulnar
2	50	Female	Left	Distal to FPL origin	Hypoplastic	FCRB > >FPL	Ulnar⟶radial
3	64	Female	Right	Distal to FPL origin partially from FDS	Hypoplastic	FCRB > >FPL	Radial
4	55	Male	Right	Distal to FPL origin	Normal	FCRB > FPL	Radial
5	59	Female	Right	Distal to FPL origin, partially from FDS	Hypoplastic	FCRB > >FPL	Radial
6	24	Male	Left	Distal to FPL origin	Hypoplastic	FCRB > >FPL	Radial
7	33	Male	Left	Distal to FPL origin	Hypoplastic	FCRB > >FPL	Radial

FCRB: flexor carpi radialis brevis; FDS: flexor digitorum superficialis; FPL: flexor pollicis longus; PQ: pronator quadratus.

## Data Availability

The data that support the findings of this study are available from the corresponding author upon request.

## References

[1] Wodd J. (1867). IV. Variations in human myology observed during the Winter session of 1866-67 at King's college, London. *Proceedings of the Royal Society of London*.

[2] Le Double A. F. (1897). *Traité des variations du système musculaire de l'homme et de leur signification au point de vue de l'anthropologie zoologique*.

[3] Yoshida Y., Yasutaka S., Seki Y. (1983). Flexor radialis profundus and palmaris profundus muscles in man. *Kaibogaku Zasshi*.

[4] Peers S. C., Kaplan F. T. (2008). Flexor carpi radialis brevis muscle presenting as a painful forearm mass: case report. *The Journal of Hand Surgery*.

[5] Kosiyatrakul A., Luenam S., Prachaporn S. (2010). Symptomatic flexor carpi radialis brevis: case report. *The Journal of Hand Surgery*.

[6] Kang L., Carter T., Wolfe S. W. (2006). The flexor carpi radialis brevis muscle: an anomalous flexor of the wrist and hand. A case report. *The Journal of Hand Surgery*.

[7] Chong S. J., Al-Ani S., Pinto C., Peat B. (2009). Bilateral flexor carpi radialis brevis and unilateral flexor carpi ulnaris brevis muscle: case report. *The Journal of Hand Surgery*.

[8] Mantovani G., Lino W., Fukushima W. Y., Cho A. B., Aita M. A. (2010). Anomalous presentation of flexor carpi radialis brevis: a report of six cases. *Journal of Hand Surgery*.

[9] Ho S. Y., Yeo C. J., Sebastin S. J., Tan T. C., Lim A. Y. (2011). The flexor carpi radialisbrevis muscle — an anomaly in forearm musculature: a review article. *Hand Surgery*.

[10] Lee Y. M., Song S. W., Sur Y. J., Ahn C. Y. (2014). Flexor carpi radialis brevis: an unusual anomalous muscle of the wrist. *Clinics in Orthopedic Surgery*.

[11] Nagata J., Kojima Y., Satomura K., Ishiko T., Ajiki T. (2016). Anatomic variations of the flexor carpi radialis brevis: a report of five cases. *The Journal of Hand Surgery*.

[12] Hosokawa T., Suto M., Tajika T., Chikuda H. (2019). Volar locking plate fixation of distal radius fracture with a flexor carpi radialis brevis and a hypoplastic pronator quadratus. *Journal of Orthopaedic Case Reports*.

[13] Bates T., Nuelle J., Pierrie S. (2019). The flexor carpi Radialis brevis: a description of an anomalous wrist flexor and surgical exposure technique. *The FASEB Journal*.

[14] Laugharne E., Power D. (2010). Surgical exposure of the distal radius in a patient with a flexor carpi radialis brevis muscle anomaly. *Journal of Surgical Case Reports*.

[15] Werntz R. L., Hadeed A. J., Cappelleti G. L., Orbay J. L. (2021). Flexor carpi radialis brevis resection for treatment of a distal radius fracture: a case report. *Journal of Wrist Surgery*.

